# Artificial intelligence–driven risk prediction of polypharmacy in older adults: current advances, clinical applications, and future perspectives

**DOI:** 10.3389/fpubh.2026.1921129

**Published:** 2026-09-03

**Authors:** Jianfeng Chen, Leyu Tao

**Affiliations:** 1 Department of Emergency, Shaoxing People's Hospital, Shaoxing, Zhejiang, China; 2 The First Hospital of Shaoxing University, Shaoxing, Zhejiang, China; 3 Department of Pharmacy, Shaoxing People's Hospital, Shaoxing, Zhejiang, China

**Keywords:** artificial intelligence, medication safety, older adults, polypharmacy, risk prediction

## Abstract

Due to the ageing of the population, multimorbidity and polypharmacy has significantly increased, causing medication safety to become a global health concern. Traditionally, medication risk assessment methods based on the counts of medications or rule-based criteria are inadequate to perceive the dynamic, individual and longitudinal nature of medication-related risks in older people. Artificial intelligence (AI) has shown promise in retrospective predictive modelling for precision medication and medication risk stratification, although prospective evidence demonstrating clinical effectiveness remains scarce. This narrative review presents the current evidence on AI-based polypharmacy risk prediction approaches in older people with a focus on machine learning, deep learning, natural language processing, graph neural network, large language models, and longitudinal trajectory modelling. We highlight some of the major data sources, predictive algorithms, clinical decision support systems and real-world applications, stressing mostly on patient-centred medication management, nurse-led interventions, transitional care and continuity of care. Furthermore, the review highlights significant challenges specifically related to model interpretability, data quality, external validation, ethical governance, privacy protection and clinical implementation which currently limits the translation of AI into routine practice. Future research needs to focus on longitudinal dynamic risk prediction and multimodal data integration. The validation of Esplanade AI by multiple centers as well as collaboration among various fields, will support better medication safety and health outcomes for Older Adults. Harmonization of contemporary AI technologies with geriatric drug use through a public health lens will provide possible guideposts for precision prevention, healthy ageing and sustainable health care delivery.

## 1 Introduction

As the global ageing process continues to accelerate, the prevalence of multimorbidity among older adults is becoming increasingly common, and polypharmacy has emerged as a significant challenge in the field of global public health. Currently, there is no universally accepted definition of polypharmacy. This fact is acknowledged in a recent report by the World Health Organization (WHO), which states: “Polypharmacy refers to the concurrent use of multiple medications. Although there is no standardised definition, polypharmacy is typically defined as the routine use of five or more medicines, and its prevalence is significantly higher among older adults who are frail, or have a heavy burden of chronic disease” (1). Studies indicate that the prevalence of polypharmacy among older adults in Europe has exceeded 30% (2), and in some long-term care facilities, it is as high as 50% or more (3). Given that older patients are characterised by reduced hepatic and renal function, altered pharmacokinetics and decreased physiological reserves, polypharmacy not only increases the risk of drug–drug interactions (DDIs) and potentially inappropriate medications (PIMs), but is also closely associated with falls (4), frailty (5), readmission (6), adverse drug events (ADEs) and increased all-cause mortality (7). Previous large-scale cohort studies have further confirmed that polypharmacy significantly increases the risk of hospitalisation and mortality in older patients (8), with a clear association between persistent polypharmacy and fall-related injuries (9). Furthermore, specific subgroups such as those with diabetes (10), chronic kidney disease (CKD) (11), cancer and cognitive impairment exhibit more pronounced high-risk medication patterns due to the complexity of long-term disease management (12).

In real-world clinical settings, older patients often present with multisystem diseases, long-term prescription adjustments and highly heterogeneous health statuses, which impose significant limitations on traditional models in terms of risk identification sensitivity, generalisability and real-time prediction. Take the Beers criteria and STOPP/START guidelines as an example. These two clinical practices are classic statistical models. In terms of statistical analysis, most of them use traditional logistic regression-type models. The weakness of this framework specification lies in the assumption of linearity of relationships. It is difficult to reflect the non-linear interactions between complex drug combinations that are often experienced in clinical practice. As a result, the risk of adverse events from potentially dangerous drug combinations is underestimated. At the same time, the construction of Beers criteria and STOPP/START is also mainly based on static rules. There is little ability to adapt and respond to dynamic changes in prescriptions and demographic characteristics of different regions. It is difficult to meet the long-term, continuous real-life risk assessment needs of the older adults (13, 14).

Over the past few years, the rapid advancement of AI and machine learning (ML) technologies has provided a novel opportunity to investigate polypharmacy risk prediction in seniors (15). AI models operating on electronic health records (EHRs), health insurance databases and prescription sequence data can assimilate demographics, disease types, drug types and overtime-use and identify complex drug interactions, predict high-risk medication use and can provide individualised risk stratification (16, 17). According to some studies, the area under the curve (AUC) of random forests (RF), gradient boosting machines (GBM) and deep learning (DL) models in polypharmacy prediction has exceeded 0.80, implying a high practical value of these models in predicting potential medication errors and supporting clinical decision-making (18, 19). At the same time, new AI technologies such as large language models (LLMs) and graph neural networks (GNNs) are being applied to the prediction of drug side effects and complex drug networks for precision medication of older adults (20).

Most past reviews have tended to focus on artificial intelligence applications pertaining to medication optimisation, clinical decision support and adverse drug events detection. However, comprehensive synthesis on the usage of AI for predicting polypharmacy risk among older adults is scarce. As such, this review differs from previous literature by focusing on risk prediction rather than medication optimisation, concerning especially older adults with multimorbidity and complex medication exposure. We integrate cutting-edge paradigms of AI, such as ML and Unsupervised Learning, Deep Learning, Graph Neural Networks, and Large Language Models. However, we do so while also critically assessing their clinical applicability, methodological limitations, and barriers to implementation in real-world medication management systems. By bridging computation with practice in geriatric pharmacotherapy, this review proposes a clinically-relevant framework describing how clinically-oriented AI may assist in medication safety, pharmacist-led medication review, deprescribing and individualised care pathways.

## 2 Methods

This review aims to synthesize the evidence on the prediction of multiple medication risks in the older adults driven by AI, and adopts the narrative review method. Based on the principles of systematic literature search, the relevant literature is sorted out, synthesized, and critically evaluated. In May 2026, we conducted a literature search in the following electronic databases: PubMed and Web of Science. The search time range was set from May 5, 2006 to May 5, 2026 to cover the latest advancements in the application of AI in the medical field, especially the development of deep learning and large language models.

The search strategy combined the following keywords and their corresponding medical subject headings (MeSH):

AI-related terms:(“artificial intelligence” OR “machine learning” OR “deep learning” OR “neural network” OR “natural language processing” OR “large language model” OR “graph neural network”).

Multimorbidity-related terms:(“polypharmacy” OR “multiple medications” OR “potentially inappropriate medication” OR “drug–drug interaction”).

Target population and outcome:(“older adults” OR “older” OR “geriatric” AND “risk prediction” OR “adverse drug event” OR “medication safety”).

Inclusion criteria: (1) The research topic is the application of AI in the risk assessment of multimorbidity in the older adults; (2) The research types include original studies, systematic reviews, and meta-analyses; (3) The language of the articles is English. Exclusion criteria: (1) Non-original comments, editorials, conference abstracts; (2) Focusing only on a single drug or a single disease, without involving multimorbidity; (3) Not providing clear performance evaluation indicators for the model. Initially, we screened the title and abstract, and read the full texts on potentially eligible literature to finally include them. As this article is a narrative review, we did not systematically assess the quality or perform a meta-analysis of the included studies, but we summarize and critically discuss key technical paths, application scenarios, and challenges.

## 3 Clinical and epidemiological basis of polypharmacy risk

### 3.1 Polypharmacy as a multidimensional medication-related risk

The threat of polypharmacy does not depend merely on the amount of medicine but also on the complex interplay which occurs between medicines, diseases and physiological functions. Older adults with multimorbidity may need long-term combination therapy of antihypertensives, antidiabetics, anticoagulants, psychotropics and analgesics. These combinations can interact through pharmacokinetic and pharmacodynamic pathways, thus heightening the risks of hypotension, hypoglycaemia, bleeding, and cognitive impairment (21). Decreased liver and kidney functions with age make drugs accumulate and cause more problems (22).

**Figure 1:**
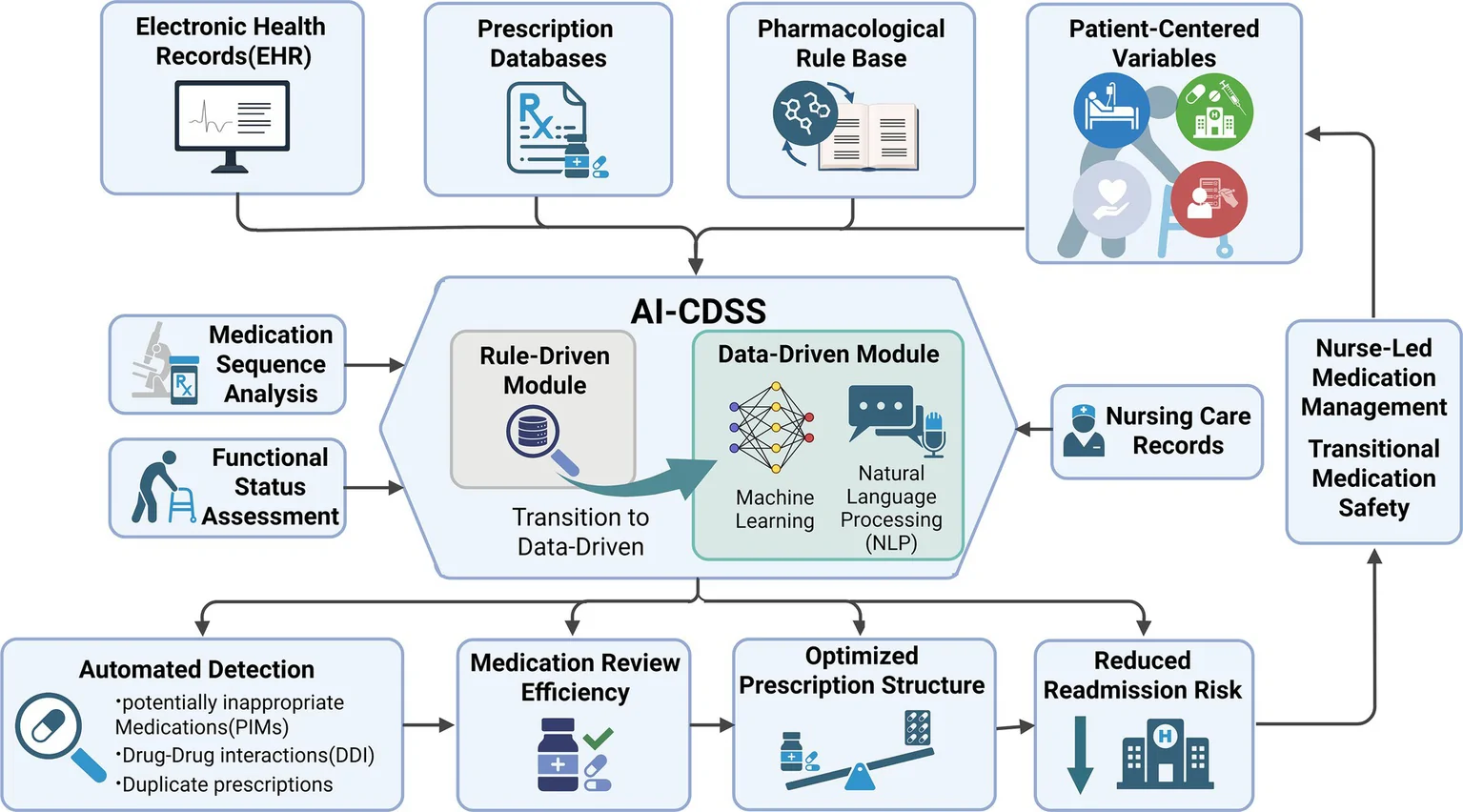
A mechanistic model of polypharmacy risk he interplay of pharmacological safety, medication burden, and patient capability.

Polypharmacy is more than just the sum of drug–drug interactions. The cumulative side-effect burden and the cumulative medication burden also matter. The use of anticholinergic, sedative, and psychotropic agents for a long time causes impaired mobility, cognitive impairment, and limitation of activities of daily living (23, 24). Complicated medication schedules may also reduce adherence and increase duplicate prescribing, self-discontinuation and medication mistake. Older adults who are cognitively impaired, have sensory deficits, or lack social support are at a higher risk of inappropriate medication use and abuse. As a result polypharmacy risk should be regarded as a multidimensional health risk in which the aspects of safety of medication, maintenance function and capacity for self-management are integrated (25, 26) (Figure 1).

### 3.2 Key prediction outcomes and risk factors

Polypharmacy has been consistently associated with several clinically important outcomes that have become major targets of medication-risk prediction models (27), including adverse drug events, falls and fall-related injuries, hospitalisation, emergency department utilisation, functional decline, reduced health-related quality of life, and mortality (28, 29).

These outcomes are influenced by multiple interacting risk factors. High-risk populations commonly exhibit advanced age, multimorbidity, frailty, renal dysfunction, cognitive impairment, polypharmacy burden, and complex therapeutic regimens. For example, patients with diabetes frequently require combined antidiabetic, antihypertensive, lipid-lowering, and antiplatelet therapy, which increases the likelihood of drug–drug interactions, hypoglycaemia, falls, and functional decline (30, 31).

Patients with chronic kidney disease are particularly susceptible to drug accumulation and toxicity because of impaired drug clearance (31). Older adults with cancer often receive anticancer therapy in addition to long-term treatment for chronic conditions, resulting in a higher prevalence of potentially inappropriate medications and adverse drug events (32).

Drug-related hazards are also modified by frailty plus psychosocial factors. Older frail adults are likely to have persistent patterns of polypharmacy with functional decline and readmission (33). Cognitive impairment, loneliness, and lack of social support may reduce adherence to medications and increase inappropriate use of medications (31). Individualised risk prediction methods that combine clinical, functional, and psychosocial information are needed due to the marked heterogeneity of these risk profiles.

### 3.3 Limitations of conventional risk assessment and rationale for AI

Over the years, traditional polypharmacy assessment has essential based on number of medication. This is often considered as five or more (34, 35). Although, the increasing evidence suggests that agent counts on their own cannot discriminate appropriately between beneficial and detrimental polypharmacy (36, 37). Some patients who undergo a number of evidence-based treatments do well, while others sustain significant harm on relatively few drugs because of potentially dangerous drug–drug combinations or interactions.

To overcome this limitation, recent studies have proposed risk stratification strategies incorporating the pharmacotherapy characteristics, clinical risk factors, and functional status (38). Longitudinal analyses indicate that different medication trajectories will be associated with different, substantially different risks of frailty, hospitalisation and mortality. This suggests that the risk attached to medication evolves over time (39).

The rising limitations have generated increasing interest in artificial intelligence–assisted approaches that can incorporate multidimensional clinical information including EHRs, prescription trajectories, laboratory results, functional status indicators, and longitudinal data. These approaches might enhance the identification of high-risk medication patterns and help individualise medication management. Current evidence mainly shows predictive performance, with few studies assessing clinical effectiveness and prospective implementation.

## 4 AI-supported approaches for identifying medication-related risks in older adults

The various AI methods tackle varying pharmacotherapeutic issues and hence are not interchangeable. Certain algorithms are more adept than others for evaluating potentially inappropriate medications and complex drug–drug interaction networks, while other algorithms focus on the analysis of longitudinal prescription trajectories, adverse drug event prediction or the extraction of medication-related information from clinical notes. Solely algorithmic attributes will not determine their performance, other things being equal, data availability, public faith, reliability, computational expense and clinical deployment. The different AI approaches discussed in this review are summarised in Table 1 for ease of comparison from the methodological and clinical pharmacy perspectives, along with their respective advantages, limitations, typical data requirements, interpretability, and clinical applicability.

**Table 1:** Comparison of the methodological characteristics and clinical applicability of major AI models for polypharmacy risk prediction.

Method	Advantages	Limitations	Typical data	Interpretability	Clinical applicability	References
Learning (RF, XGBoost)	High prediction performance, handling of nonlinear relationships, and relatively easy model interpretability	Manual feature engineering is required, and it is difficult to handle the original high-dimensional data (such as text and images)	Structured tabular data (such as demographics, diagnoses, prescription quantities)	Medium-high (explainable through SHAP, LIME)	Risk stratification, PIM/ADE prediction, identification of key risk factors	(14, 15, 67, 68)
Deep learning (LSTM, Transformer)	Automatic feature extraction, skilled in handling time series and sequence data	The “black box” feature requires a large amount of data and incurs high computational costs	Vertical prescription sequence, EHR timeline data	Low	Dynamic risk prediction, modelling of complex medication trajectories	(50–52)
Graph neural networks (GCN, GAT)	Good at modelling relationships (such as drug–drug, drug-disease), it can perform link prediction	The graph is highly complex, highly dependent on the quality of the graph, and has poor interpretability	Drug molecular structure, knowledge graph, DDI network	Low	New drug DDI prediction and discovery of rare high-risk drug combinations	(74, 75)
Large Language Model (LLM)	Strong semantic understanding, capable of handling unstructured text, and strong knowledge integration capabilities	There is a risk of “hallucination,” unstable reasoning, and prominent ethical and privacy issues	Clinical texts (nursing records, discharge summaries), medical knowledge base	Medium (can be enhanced by RAG)	Patient risk profiling, extracting risk information from text, assisting clinical decision-making	(53–55)

### 4.1 Clinical targets for AI-based medication risk prediction

Identifying high-risk medication combinations and PIMs is a fundamental challenge in the medication management of older adults with multimorbidity. Older people are often prescribed multiple drugs together leading to increased likelihood of clinically significant DDI’s, cumulative drug burden and ADE’s (13). Alternative medication review strategies such as Beers Criteria, STOPP/START criteria, and rule-based clinical decision support systems (14), are still useful for detecting inappropriate prescribing (15); However, these methods are mainly rule based and often cannot identify complex interactions between multiple medications, patient characteristics, and changing patterns of prescribing over time.

To address these limitations, Before model development, heterogeneous electronic health record (EHR) data are typically transformed into structured numerical representations suitable for machine learning (40). Categorical variables, including medication classes, diagnoses, and comorbidities, are commonly encoded using one-hot encoding or learnable embedding vectors, whereas continuous laboratory measurements are normalised or standardised (41). Because longitudinal EHR data are often sparse and irregularly sampled, missing observations are generally addressed through multiple imputation, masking strategies, or temporal aggregation, enabling downstream models to learn robust medication-related risk patterns from incomplete clinical records (42). Model selection should be driven by the characteristics of the available clinical data rather than algorithmic complexity alone. Structured tabular EHR data are commonly analysed using tree-based ensemble methods, whereas large-scale longitudinal multimodal datasets are more amenable to deep learning architectures capable of modelling complex temporal dependencies (40). AI has increasingly been explored as an auxiliary decision-support tool for identifying high-risk medication combinations and supporting medication safety management (43). By integrating multidimensional information from EHRs, longitudinal prescription sequences, disease profiles, laboratory findings, functional assessments, and medication histories, Preliminary retrospective studies suggest that AI models may be capable of identifying medication-related risk patterns that are less readily recognised using conventional rule-based approaches, although prospective clinical validation remains limited (16).

Studies indicate that DL- and GNN-based models can effectively characterize complex DDI networks that are difficult to detect using traditional rule-based systems while improving the identification of medication combinations associated with ADEs (44).

However, current evidence primarily demonstrates improvements in predictive performance rather than definitive improvements in clinically meaningful medication-related outcomes (45). Most available studies are retrospective and evaluate model discrimination using statistical indicators rather than patient-centred endpoints, highlighting the need for prospective implementation studies before widespread clinical adoption (46).

According to a clinical pharmacy perspective, the main benefit of AI-based identification of PIMs and DDIs is to make the medication review process more efficiently and prioritised while not replacing judgement. In everyday geriatric medication management, pharmacists need to assess complex medication regimens in terms of their clinical indication, treatment goals, renal and hepatic function, frailty status, life expectancy and patient preferences. Risk scores derived from AI may help pharmacists quickly screen a large number of people and identify those most likely to benefit from comprehensive review (47).

For instance, AI systems might flag patients with high-risk medication combinations, an excessive medication burden, or a clinically relevant pattern of DDI prior to pharmacist-led medication reconciliation. AI outputs should be taken as a useful information and not as a prescribing advice. The pharmacist will continue to validate alerts generated by AI, distinguishing between clinically appropriate treatment and inappropriate prescribing, and coordinating medication optimisation with doctors and nurses.

Accordingly, the involvement of artificial intelligence in product information management and drug–drug interaction management should follow a human-in-the-loop model wherein AI detects risk and pharmacist contextual clinical evaluation decision-making.

### 4.2 AI for predicting adverse drug events and medication adherence

Predicting ADEs and identifying patients at risk of poor medication adherence remain major challenges in the pharmacological management of older adults with polypharmacy (20). Older people are at serious risk of harm from medication because they experienced a lot of physiological changes due to age and they have other ailments, clogged medicines and many transitions of care. Health providers’ traditional practice and prescription rule-based methods of risk assessment employ isolated clinical variables. Hence, they often do not capture the complex interaction of medicines, comorbidities, lab results or any behavioral factor that leads to non-adherence (NA) and ADEs (48).

To address these limitations, there has been increased exploration of AI as an adjunct clinical decision-support tool for predicting medication-related risks (49). AI models can be developed that identify complex patterns associated with ADEs and/or medication non-adherence with integration of associations from the multidimensional information in electronic health records, laboratory indicators, trajectories of prescriptions, clinical notes, and patient reported information (48).

Long short-term memory (LSTM) models or Transformer-based architectures are deep learning models that have superior advantages for modelling longitudinal medication exposure and clinical changes over time (50). LSTM networks use input, forget and output gating mechanisms to keep or discard previous clinical information selectively so that the long-term dependencies in time can be captured in prescription sequential data. LSTM models learn the temporal evolution of medication-related risk by treating each medication refill, medication change, or visit to the hospital as a time step in order (51, 52). In the course of model training, backpropagation through time (BPTT) is often used to optimise the parameters cross-entropy loss is typically used on the classification tasks to minimize prediction errors on future clinical time-points. Rather than having recurrent neural networks which have sequential recurrence, Transformer architectures implements multi-head self-attention mechanisms enabling it to model relationships simultaneously among medications, lab results, diagnoses, and clinical events during the whole observation period (53). The data is arranged in their order rather than the actual data points in the original architecture. In doing so, the positional encoding captures the temporal order of the data even when they are computed in parallel. The use of positional encoding allows Transformers to appropriately model irregularly sampled longitudinal EHR data. Thus, the architecture can accurately model long-range medication trajectories. These methods facilitate the ongoing surveillance of medication-related risks via the sequential prescription records and patient data, which will promote the early identification of those who could benefit from a medication review or from closer follow-up (44).

The output of LLMs are contextual embeddings that can capture complex semantic relationships in clinical narratives and are generally pretrained with self-supervised next token prediction objectives (54). These embeddings can subsequently be combined with structured EHR variables to facilitate adverse drug event detection, medication reconciliation, and automated extraction of medication-related information from discharge summaries and clinical notes (55, 56). In addition, natural language processing (NLP) and LLMs have shown potential for extracting clinically relevant medication information from unstructured electronic health records, discharge summaries, and clinical narratives (57). These technologies may facilitate the detection of undocumented ADEs, medication discrepancies, and adherence-related issues that are often overlooked by structured databases (58, 59), However, current evidence supporting the application of large language models in medication safety remains largely limited to proof-of-concept studies, retrospective evaluations, or early implementation reports, with few large-scale prospective clinical validation studies currently available (60).

From a clinical pharmacy perspective, AI-generated risk predictions should be interpreted as supportive information rather than autonomous prescribing recommendations (61). AI-assisted alerts may help pharmacists identify patients at high risk of medication-related harm, prioritise comprehensive medication reviews, monitor adherence during long-term pharmacotherapy, and optimise medication reconciliation during transitions of care (26, 62). Such applications may also facilitate shared decision-making by enabling more timely medication counselling and individualised follow-up for vulnerable older adults (63).

Despite encouraging predictive performance reported in recent studies, current evidence primarily focuses on model discrimination rather than clinically meaningful patient outcomes (64). Most published studies are retrospective and evaluate statistical performance using metrics such as the AUC, sensitivity, and specificity. It is still unknown whether we could use AI-based prediction of ADEs and medication adherence for early risk identification. However, whether they could reduce medication-related hospitalisation, improve functional status, and enhance health-related quality of life, all need prospective validation (65). Therefore, prospective implementation studies and pragmatic clinical trials are required before these technologies can be routinely incorporated into pharmacist-led medication management (66).

The proactive prevention of adverse drug events and non-adherence may become possible through AI-driven prediction of risks and subsequent enhanced medication management. In clinical pharmacy practice, risk predictions generated by artificial intelligence may help pharmacists recognize which older individuals need enhanced medication counselling, adherence assessment or follow-up monitoring. For example, due to polypharmacy, renal insufficiency, a prior ADE-related hospitalisation, and/or a complex medication regimen, patients may be targeted for pharmacist interventions (educating the patient on medications, supporting adherence, and/or making monitoring recommendations). In addition, natural language processing and LLMs could help pharmacists extract drug-related data from unstructured clinical documents, such as discharge summaries, nursing records, and patient communication notes, thereby enhancing medication reconciliation and discharge counselling efficiency. Nonetheless, AI-driven predictions should not be seen as definitive clinical conclusions. Pharmacists should be the last reviewers of AI outputs by verifying the output of the algorithm with their clinical knowledge, direct patient assessment and shared decision-making principles. The input states that the interventions which are supported by AI related to the medications are clinically and patient-centred. The interpretation by the pharmacist is very crucial.

### 4.3 AI for dynamic medication risk stratification and longitudinal monitoring

Medication-related danger is not static but dynamic. This is especially the case for older individuals with multimorbidity and polypharmacy. During hospital stays, transitional care, and long-term disease management, a patients’ medicinal regimens change continuously which alters the risk related to medication (67). Changes in combinations of drugs, renal function, frailty, laboratory results and adherence to medicines may significantly change a person’s risk of an adverse drug event over time. Conventional risk assessment tools have limited ability to reflect evolving medication-related risks over time, as they typically provide a single cross-sectional estimate based on baseline characteristics. Dynamic risk stratification approaches informed by longitudinal clinical data are needed (63).

Conventional logistic regression models presume a linear association between the predictor variable and the log-odds of the clinical outcome unless interaction or nonlinear terms are stated explicitly (68). However, the risks associated with medications in older adults often arise from complex interactions, including multiple medications, comorbidities, shrinkage of physiological reserve, and functional decline, which make it difficult to meet those assumptions (65).

Tree-based ensemble learning algorithms such as Random Forest and Extreme Gradient Boosting (XGBoost) partition the feature space recursively to clinically meaningful thresholds and combinations of patient characteristics (68). Each recursive partitioning step separates patients into increasingly homogeneous sub-groups on the basis of predictor values that maximally gain information or minimize node impurity in a way that threshold effects emerge naturally without requiring prespecified interaction terms (69). From a clinical pharmacy viewpoint, active risk stratification offers pharmacists. These models, instead of estimating a single linear coefficient for each predictor, automatically detect non-linear threshold effects and higher-order interactions that would otherwise go undetected. An example of this is a decision tree that first distinguishes patients by age (>75 years), then by number of concomitant medications (>10 drugs), and finally by those receiving high-risk medications (e.g., benzodiazepines, anticoagulants), resulting in more refined medication-related risk strata.

By adapting to this hierarchical categorization, AI models can identify “tipping points” of clinical significance with regard to medication safety. The risk of falling due to sedative use may go up in a robust older adult but may not go up as much to frail older adults taking several CNS (central nervous system) active drugs (70, 71). Tree-based models are especially appropriate for the identification of these threshold effects since they place no prior assumptions on either interactions among variables or linearity of the effects and automatically capture nonlinear effects from longitudinal clinical data (72).

Several deep learning architectures beyond traditional tree-based models have been developed to model the temporal and relational complexity of medication-related risk. RNNs and LSTM networks are updating hidden states across clinical visits due to which they can capture sequential dependencies in the longitudinal prescription records and EHR trajectories (73). The use of multi-head self-attention mechanism in place of recurrent computation in transformer architectures make it possible to efficiently model long-range dependency among the medications, diagnoses, lab tests and clinical events throughout the entire period of observation (74). Temporal GNNs also integrate graph-based encoding of patients, medications, diagnoses, and lab variables, enabling simultaneous modelling of temporal evolution and complex networks of medication interactions which are difficult to model with conventional sequential models (75). As such, alterations to medication regimens may lead to dynamic updates of risk estimates related to medication. However, most evidence currently available comes from retrospective modelling studies using EHR-derived data rather than from prospective clinical implementation (48).

GNNs further extend these capabilities by representing patients, medications, diagnoses, and laboratory variables as interconnected nodes within a heterogeneous clinical graph (76). Through iterative message-passing operations, node representations are continuously updated by aggregating information from neighbouring nodes, enabling the model to capture higher-order medication interactions and complex disease–drug relationships that are difficult to represent using conventional tabular learning approaches. This graph-based representation is particularly advantageous for modelling polypharmacy, where medication-related risks frequently arise from interconnected drug–drug, drug–disease, and patient-specific relationships rather than isolated predictors (43).

From a clinical pharmacy perspective, dynamic risk stratification provides pharmacists with continuously updated information regarding medication-related risk rather than a single static prediction (77). Such systems may facilitate prioritisation of medication reviews, optimisation of monitoring intensity, early identification of patients requiring deprescribing, and timely medication reconciliation during transitions of care (78). Importantly, AI-generated predictions should be interpreted as decision-support information that complements multidisciplinary clinical judgement rather than replacing pharmacist-led decision-making (79).

Despite promising predictive performance, current studies predominantly evaluate statistical discrimination rather than demonstrating improvements in clinically meaningful outcomes (80). Most published evidence is retrospective, and prospective implementation studies examining workflow integration, pharmacist acceptance, medication appropriateness, and patient-centred outcomes remain limited (81). Therefore, pragmatic clinical trials are required before dynamic AI-based medication monitoring can be routinely integrated into medication management programmes (82).

The traditional approaches to the assessment of medicine typically provide a snapshot assessment. But dynamic AI-based Risk Stratification will allow for the continuous assessment of medicine-related harms over time. In terms of pharmaceutical care, this capability could facilitate on-going medication management through identification of changes in risk profile requiring a re-evaluation.

As an illustration, a rise in risk scores derived from the AI systems can indicate the emergence of issues like medication burden, disease progression, functional decline, or cumulative adverse events. Pharmacist-initiated medication review and reconsideration of the necessity of medication follow-up and deprescribing when clinically indicated. AI can serve as a platform in multidisciplinary geriatric care to support decision making by providing access to the same information to pharmacists, physicians, nurses, and other health care providers. Although pharmacists may interpret risks and recommend optimisation, physicians may assess doctors’ therapeutic necessity, clinical indication, while nurses may help with information about adherence, functional change and self-management capacity. This collaborative model could make the most of artificial intelligence’s clinical utility with human responsibility.

### 4.4 Evidence synthesis of current AI-based medication risk prediction studies

As can be seen from Table 2, the current AI-based approaches for medication-related risk prediction and safety assessment for older adults have so far shown considerable predictive power on various clinical objectives. These include polypharmacy risk, adverse drug event prediction, drug–drug interaction detection, and medication trajectory analysis. When available, reported discrimination performance is largely in the acceptable range with many studies showing moderate-to-high predictive accuracy. Comparison of model performance is difficult because the prediction targets, study populations, data sources and evaluation frameworks differ greatly from study to study.

**Table 2:** Representative evidence on AI-based medication risk prediction and safety assessment in older adults.

Study (year)	Population/data source	Prediction target	AI approach	Validation strategy	Key findings	References
Santos-Perez et al. (2018)	Older adults; clinical dataset	Polypharmacy risk	Machine learning	Model development with internal performance evaluation	High-risk polypharmacy identified	(18)
Sirois et al. (2021)	Quebec population database	Medication exposure patterns	AI analytical framework	Methodological framework development; predictive performance not evaluated	Medication patterns characterised	(17)
Elhosseiny et al. (2025)	SHARE cohort	Polypharmacy risk	Data-driven AI	Population-based predictive modelling and model performance evaluation	Multidimensional risk prediction	(16)
Elhussein et al. (2025)	UK & Netherlands cohorts	Medication trajectories	Joint latent class model	Longitudinal observational analysis	Persistent polypharmacy associated with mortality	(39)
Frydenlund et al. (2025) (ADAPTiP tool)	Geriatric patients	ADE/ADR risk	ADAPTiP prediction model	Model development and validation in geriatric populations	High-risk patients identified	(44)
Zitnik et al. (2018)	Drug interaction network	Polypharmacy side effects	Graph convolutional network	Network-based prediction evaluation	Drug interaction effects predicted	(43)
Huang et al. (2023)	Drug interaction databases	Drug–drug interactions	Phar-LSTM	Evaluation using DDI prediction/extraction metrics	Improved DDI prediction	(52)
Carrasco-Ribelles et al. (2023)	Longitudinal AI prediction studies	Methodological evaluation	Systematic review	Methodological appraisal of published AI prediction studies	Validation and implementation gaps identified	(51)

Notably, most prior studies have conducted through retrospective observational datasets such as electronic health records, administrative databases, and population-based cohorts. Although these data sources can be used to develop large-scale models, retrospective designs may capture past rather than optimal prescribing, leading to selection bias, confounding, and biases linked to the healthcare system. As a result, superior predictive performance may not translate into clinical impact or enhanced medication outcomes.

Moreover, there is a lack of external validation in other AI-based medication risk prediction studies. There are differences in prescription behaviour, coding of medications, access to health care facilities and population characteristics that may affect model transportability between institutions and countries. Moreover, while most research studies report discrimination metrics such as AUROC, few provide calibration measures, decision-curve analyses, or prospective evaluations of clinical utility.

These findings suggest that the existing evidence base is sufficient to support the capacity of AI to predict medication-related risks. However, current evidence is insufficient to support the use of AI to enhance medication safety outcomes in routine geriatric care. Subsequent research should therefore make systems that have been validated externally a priority as well as standardisation for dependable reporting frameworks.

**Figure 2:**
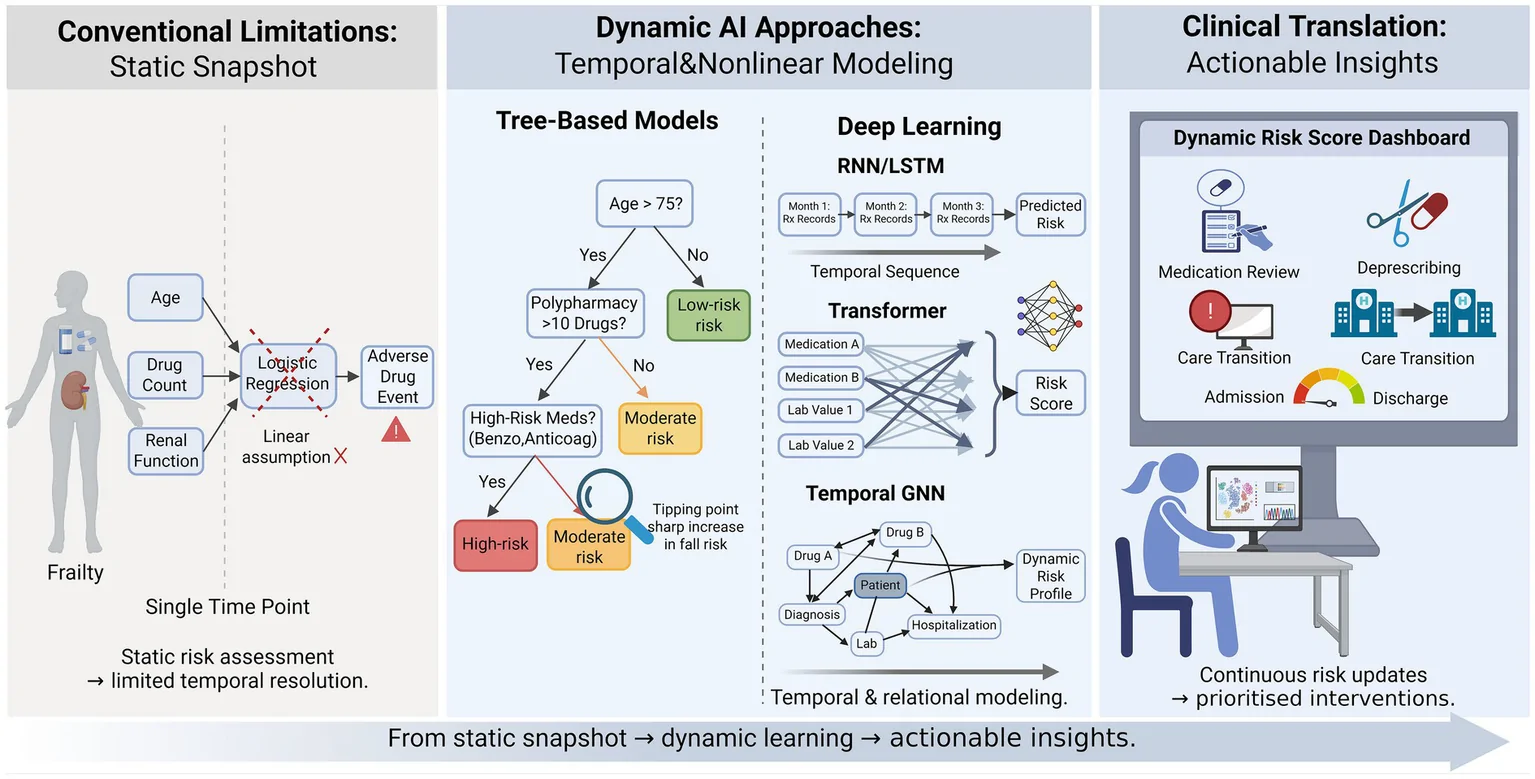
AI for dynamic medication risk stratification and longitudinal monitoring.

Among the studies in Table 2 with a clinical focus, the majority consisted of retrospective observational analyses, with only a minority indicating independent validation beyond the development dataset. In addition, calibration assessment and prospective evaluation of clinical utility were rare (Figure 2).

## 5 Empirical applications and effectiveness evaluation

The professional synthesis information provided in Table 2 indicates the important distinction between predictive capability and clinical effectiveness. Consequently, while approaches powered by artificial intelligence could enhance the identification and prioritisation of potential harmful effects of medication, careful thought must be given to workflow integration, clinical accountability and prospective outcome evaluation, if they are to be successfully translated into pharmaceutical care for older adults.

### 5.1 AI-enabled clinical decision support systems and pharmacist-led medication management

With the advancement of AI and clinical informatics technologies, AI-based CDSS have emerged as potential decision-support tools for managing polypharmacy in older adults. Currently, most CDSS rely primarily on EHRs, prescription databases and pharmaceutical rulebases to enable the automated identification of PIMs, DDIs and duplicate prescriptions (45, 46). Systematic reviews indicate that AI-CDSS may improve the efficiency of medication reviews and assist clinicians in identifying potentially inappropriate prescribing. However, current evidence mainly demonstrates improvements in process measures rather than definitive improvements in patient-centred clinical outcomes (45).

In recent years, with the advancement of ML and NLP technologies, Recent retrospective studies suggest that AI-CDSS is gradually evolving from traditional rule-based systems toward data-driven decision-support approaches. However, evidence demonstrating improvements in real-world medication safety outcomes remains limited. Research suggests that ML-based CDSS can overcome the limitations of traditional static rule-based systems and more effectively identify complex drug combinations and latent high-risk prescribing patterns (15, 20). Particularly among older patients with multiple co-morbidities, AI-CDSS may support patient-centred medication risk assessment and clinical decision-making by integrating multidimensional clinical information. Nevertheless, its effectiveness depends on clinician interpretation, workflow integration and local healthcare contexts (83). To improve the clinical reliability of AI-assisted decision support, model development should not rely exclusively on data-driven optimisation but should also incorporate validated pharmacotherapeutic assessment tools as clinically informed prior knowledge (61). Well-established tools, like the Medication Appropriateness Index (MAI), Beers Criteria, STOPP/START criteria, and Anticholinergic Cognitive Burden (ACB) scale, may be included as weighted clinical features or hard constraints during model development (67, 84). These knowledge-guided approaches allow AI systems to segregate between statistically valid prescribing patterns and clinically invalid medication use. In other words, the use of such approaches would lower the chances of generation of technically valid but clinically unvalid recommendations by AI systems. Rather than replacing guideline-based medication assessment, AI models should augment pharmacist-led medication review by integrating both empirical data and established geriatric pharmacotherapy principles (26).

Furthermore, research in the nursing field has highlighted that AI-CDSS also holds significant application value in nurse-led medication management and transitional medication safety (85). Several studies have indicated that enhancing decision support systems with patient-centred variables such as nursing assessments, ADL status, medication adherence and care dependency may improve the identification of high-risk older patients and enable targeted transitional care, but whether these approaches reduce medication discrepancies or readmissions requires prospective validation (86). Consequently, future AI-driven CDSS should integrate both validated pharmacotherapeutic assessment tools and data-driven prediction models to achieve clinically acceptable and evidence-based medication decision support.

AI in clinical decision support systems, or AI-CDSS, is one of the more promising avenues to introduce artificial intelligence into medications management workflow. Unline other rule-based systems that depend mainly on some criteria such as Beers Criteria, STOPP/START criteria, or medication interaction databases, AI-CDSS can consider multidimensional patient information and detect complex patterns of medication-related risks.

Importantly, the AI-CDSS should complement the established pharmaceutical benefit assessment but not replace it. The MAI (Medication Appropriateness Index), Beers criteria, and STOPP/START criteria are examples of tools with clinical validity for assessing medication appropriateness. These tools can potentially benefit from AI models, which may serve to prioritise patients at high-risk and/or identify patterns that rules may miss.

**Figure 3:**
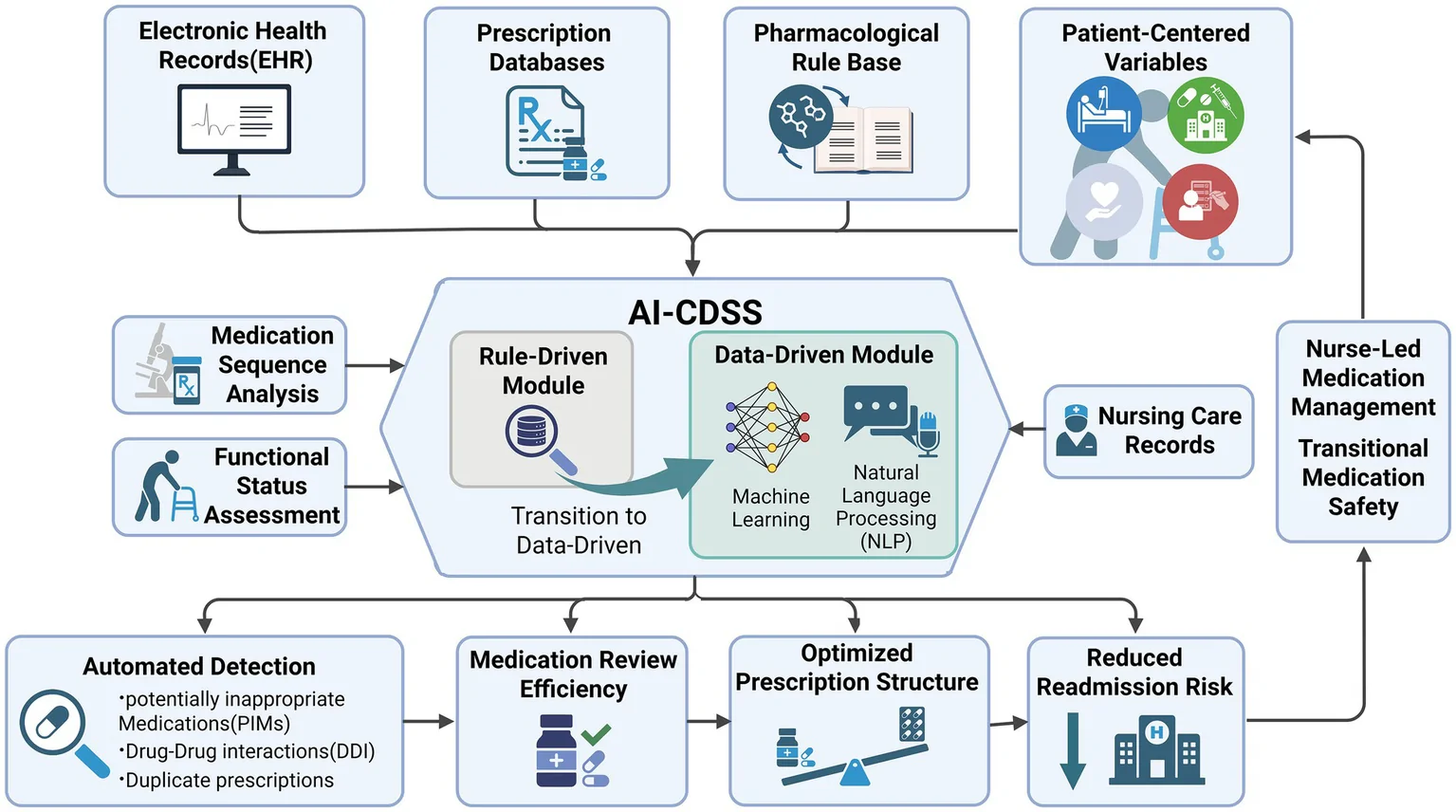
Application workflow of the AI-CDSS in nurse-led transitional medication management and safety optimization.

Implementation wise pharmacist’s play important roles in AI-CDSS supported medication management. First off, pharmacists are clinical evaluators who determine whether the alerts generated by AI are clinically relevant and identifies real medication risks from appropriate treatment choice. Moreover, the second role refers to an intervention coordinator who communicates recommendations to physicians as well as the nursing teams. Thirdly, patient-centred educational services are delivered by pharmacists to enhance understanding of and adherence to therapies. Pharmacists can look at the risk scores generated by AI to identify the older adults with recent changes to their medicines, complex regimens, renal impairment or previous drug events, before daily ward rounds. Subsequently, high-risk patients can be referred for medication reconciliation, deprescribing assessment and adherence counselling. Throughout this workflow, clinicians are directed to patients who would afford maximum benefit by the AI, leading to improved efficiencies. Final medication decisions, however, remain dependent on clinical judgement (Figure 3).

### 5.2 Regional case studies

#### 5.2.1 Regional risk maps for polypharmacy in community-dwelling older adults in China

Currently, Several retrospective modelling studies have explored AI-assisted prediction of polypharmacy risk in older adults is being explored in practice across multiple countries and regions, gradually forming data application models with regional characteristics.

In China, some studies have utilised real-world data from community-dwelling older adults, integrating variables such as demographic characteristics, the number of chronic conditions, long-term prescriptions, functional status and medication adherence to construct nomogram models for assessing the risk of polypharmacy in older adults, thereby enabling early screening of high-risk groups. Research indicates that the predictive performance of these models is significantly improved when variables relating to the burden of chronic disease, long-term prescription trajectories and functional status are incorporated (44) Concurrently, primary care institutions in China have in recent years progressively integrated nurse-led medication education with community-based chronic disease follow-up, providing a practical foundation for continuous medication safety management.

#### 5.2.2 Stratification of longitudinal prescription trajectories in Canada

In the Quebec region of Canada, research on polypharmacy trajectories based on long-term longitudinal prescription data has been conducted using chronic disease monitoring systems and health insurance databases. Through latent class analysis (LCA) and multi-trajectory modelling, these studies have dynamically stratified different long-term medication patterns and revealed a significant association between persistent high-complexity polypharmacy trajectories and the risks of mortality, hospitalisation, and functional decline (39). The researchers further proposed that precise risk stratification and dynamic medication safety management for older patients may benefit from consideration of long-term health risks rather than simply the number of medications (83).

#### 5.2.3 Long-term medication management for the very older adults in Japan

As one of the world’s most aged societies, Japan has attracted widespread attention for its research into the management of long-term prescriptions for the older adults. Some studies, based on national claims databases, have analysed long-term medication patterns among the very older adults (aged ≥85 years), finding that the long-term combined use of psychotropic drugs, sedatives and hypnotics, and anticholinergic drugs is relatively common and is closely associated with an increased risk of falls, functional decline, and dependence on long-term care (87). Furthermore, Japanese researchers have emphasised the need to implement individualised deprescribing strategies for older patients, incorporating the concepts of frailty assessment and functional preservation, in order to mitigate the long-term impact of cumulative medication burden on overall health status (47).

### 5.3 Evidence from AI-based medication risk prediction studies

#### 5.3.1 Efficiency and accuracy of medication risk identification

In recent years, a large number of studies have preliminarily confirmed the value of AI tools in the risk management of polypharmacy in older adults. Systematic reviews suggest that AI tools may support the efficiency of identifying PIMs, DDIs and ADE risks, whilst potentially reducing the time burden associated with traditional manual review; however, their impact on patient outcomes requires further evaluation (14, 46). Particularly among older patients with complex chronic conditions, ML models have demonstrated comparable or superior predictive discrimination to traditional rule-based methods in retrospective validation studies (50).

#### 5.3.2 AI-supported prescribing optimisation and potential clinical implications

Some retrospective implementation studies suggest that AI-CDSS may contribute to reducing inappropriate prescribing. Research shows that AI-based medication review interventions have been associated with improved identification of potentially inappropriate medications and changes in prescribing patterns in retrospective studies; however, whether they reduce medication-related harm requires prospective validation (47, 88). At the same time, AI intervention models combined with nurse-led medication management may support medication adherence, self-management capabilities and transitional medication safety (85, 89).

#### 5.3.3 Risk prediction performance in nursing settings

In nursing settings, AI risk prediction models are increasingly being investigated for the prediction of falls, functional decline and readmission risks. Some studies have found that incorporating ADL status, care dependency and medication self-management ability into AI models can significantly improve predictive capabilities regarding frailty progression, long-term care needs and readmission risks (45, 47). Furthermore, several studies have suggested that ML-based post-discharge fall prediction models may facilitate the early identification of high-risk older patients and support transitional care planning (47).

In actual nursing practice, nurse-led medication management has gradually evolved from traditional, single-point medication education into a comprehensive medication safety intervention covering the entire process from hospitalisation through the post-discharge transition period to continuous home care. By implementing medication reconciliation, patient-specific medication education, transitional medication counselling and post-discharge follow-up, the nursing staff can actively detect the discrepancies and non-adherence with medication and risks of potential ADEs while enhancing the patients’ medication self-management capacity and involvement of caregivers (89). Nursing teams can partner with pharmacists and prescribers on long-term medication reviews and deprescribing assessments to achieve continuous medication optimisation. With the aid of AI-based risk prediction, nurses may further use the results of model-based identification to implement targeted stratified interventions in patients with frailty, high medication burden or cognitive decline. These AI-assisted risk stratification results may may assist nurses prioritise high-risk patients and support targeted interventions. However, whether these strategies ultimately improve long-term patient-centred outcomes remains to be established through multicentre prospective studies.

Consequently, the application of current AI tools has gradually expanded from mere “medication risk identification” to patient-centred risk assessment and support for long-term, continuous care.

### 5.4 Evidence gap between predictive performance and clinical effectiveness

Although numerous studies have reported encouraging predictive performance for AI-based models in identifying potentially inappropriate medications, adverse drug events, and high-risk prescribing patterns, it is important to distinguish predictive accuracy from clinical effectiveness. Most currently available evidence is derived from retrospective observational studies or secondary analyses of electronic health records, with model performance primarily evaluated using statistical metrics such as the AUC, sensitivity, specificity, and F1-score. Though useful in assessing discrimination ability of the models, they do not necessarily demonstrate improvements in patient-centred clinical outcomes. Currently, there is limited evidence that AI-assisted, medication management reduces the risk of death, functional decline, hospital readmissions, or improves health-related quality of life. In addition, factors such as acceptance by clinicians, integration into workflows, how implementation is handled and collaboration between areas will impact the clinical effect of an AI system. Therefore, future research should prioritize multicentre prospective studies, pragmatic clinical trials, and implementation research to determine whether AI-assisted risk stratification can produce meaningful and sustainable improvements in real-world clinical outcomes. These evidence gaps also explain many of the technical and implementation challenges discussed in the following section.

#### 5.4.1 Distinguishing predictive performance from clinical effectiveness

Throughout this review, we have made a clear distinction between predictive performance and clinical effectiveness as they represent different stages of evidence development. Predictive performance is the statistical ability of an AI model to distinguish people with different levels of medication-related risk. It can be evaluated with respect to the AUROC, sensitivity, specificity, calibration or F1-score. On the other hand, clinical effectiveness refers to whether implementation of an AI-based intervention results in demonstrable improvements in patient-centred outcomes, including reduced ADEs, medication-related hospitalisations, functional decline or enhanced health-related quality of life.

The majority of studies that exist have assessed predictive performance using retrospective observational data, such as electronic health records, administrative claims and prescriptions databases, according to the evidence reviewed in this work. Multiple models have shown good discrimination to identify those patients who are at increased risk of harm from polypharmacy. However, the results should not be interpreted to mean that the implementation of AI leads to better outcomes in the medication safety arena.

At present, there is not enough of prospective evidence to suggest that AI-based polypharmacy risk prediction results in reduced incidence of ADEs (adverse drug events), improved medication appropriateness or enhanced clinical outcomes in older adults. The absence of such evidence does not imply a failure of AI approaches, but instead reflects the infancy of this research field in the translational stage. Subsequent research ought to de-emphasise retrospective model development to increase future prospective validation, pragmatic clinical trials and implementation science approaches to determine whether AI-supported medication management can lead to real benefits in the health service.

It is time to regard AI as an augmentation technology for risk stratification and clinical decision support instead of a sufficiently established intervention with demonstrated effect on patients’ outcome.

## 6 Challenges and limitations

### 6.1 Technical aspects: methodological challenges in AI-based medication risk prediction

Even though AI-based approaches yield promising results in predicting medication-related risks among older adults, there are several methodological challenges that hinder their clinical translation. In fact, these problems aren’t just technical issues; they are actually revealing the limitations of how risks related to medication are currently modelled and interpreted.

#### 6.1.1 Retrospective data and the risk of learning historical prescribing patterns

Retrospective datasets have been used to develop multiple contemporary polypharmacy risk prediction AI models. The EHR, the administrative claim, and prescription databases. While the information provided is extensive and real-word, the retrospective character of these data has important methodological limitations.

Historic healthcare data incorporate previous prescribing behaviour, not optimal pharmacotherapies, raising concerns, a systematic analysis Involving Secondary Data Reveals that either directly addressed the issue or the authors discussed. These AI models, through their training on these data, may end up learning about the clinical practice existing for the type of condition being modelled, when this is actually not the case, due to a lack of data. Thus, a model with high predictive accuracy has not necessarily learned the optimal medication decision rules, but rather it approximates historical clinical practice patterns.

The differentiation between the terms ‘treatment’ and ‘exposure’, as used in the context of pharmacotherapy of older patients, is an important one. Treatment must take into account much more than simply the exposure to an agent in the older adults. Other factors involved include treatment goals, life expectancy, frailty, functional capacity, and patient preferences. Future AI models must augment their LLM capabilities with clinically validated knowledge sources, including Beers Criteria, STOPP/START criteria, medication appropriateness assessment tools, and medication restrictions as defined by experts, to mitigate the risk of duplicating suboptimal prescribing practices.

#### 6.1.2 Limited external validation and model transportability

Insufficient external validation of existing AI models is another major limitation. Research is developed and evaluated in a single healthcare system, which raises questions about the generalisability of the research.

The model transportability challenges arise from various sources. There are large variations in prescribing behaviours across countries, healthcare systems and institutions. When a medication pattern is deemed high risk in one situation, it will have different clinical implications elsewhere. As a second point, variation in medication coding systems (e.g., ATC vs. national prescription coding system) might differ how features are represented and might affect model performance. The variance in demographic factors can affect the outcome of exposure to a drug and its outcome. Aspects like access to medical care, health insurance coverage.

Furthermore, there is inconsistency in the reporting standards of AI predictive models. Although initiatives such as TRIPOD-AI were created to improve accountability and reproducibility, compliance remains patchy. The independent assessment of existing models and their comparison is hindered by reporting model development methods, calibration performance, handling of missing data and external validation.

#### 6.1.3 Explainability, accountability, and ethical considerations

The second major AI challenge in drug risk prediction is explainability. Past discussions usually characterize interpretability as a technical problem linked to the “black-box” nature of advanced algorithms. Clinical pharmacotherapy, however, explainability, not just technical transparency, but rather accountability/ethics more broadly speaking.

When AI creates recommendations for medicine risk, clinicians cannot know whether these recommendations accord with their own clinical reasoning. As well, it is difficult for clinicians to know whether they could appropriately justify their decisions when the reasons are not made clear. It is important in case AI-assisted decisions influence deprescribing, continuing the medication, or risk communication with patients.

Consequently, explainable AI must provide the rationale of clinical meaning not only statistical justification and feature importance but also meaningful for professional accountability. AI systems must be able to have transparent pathways so that recommendations may be understood, assessed, and appropriately challenged by a pharmacist and a clinician.

### 6.2 Barriers to clinical integration

#### 6.2.1 Workflow integration and alert fatigue

Integrating the AI-based medication risk prediction into routine practice requires careful attention to the clinical workflow. Previous experiences with conventional clinical decision support systems have demonstrated that excessive alerts can result in alert fatigue; progressively diverting the attention of clinicians and heightening the risk of overlooking important alerts (45).

The issue could be exacerbated by AI systems making more frequent low-value predictions without proper prioritization. Implementation must therefore consider more than model accuracy but also the design of outputs that are actionable in terms of alert thresholds, risk stratification categories and embedding in existing medication review workflows.

Instead of contributing to the increasing number of alerts, clinically useful AI systems should guide healthcare professionals to identify the patient who needs help first. For instance, AI-generated medication risk scores can support pharmacists in identifying and prioritizing older adults with high medication burden, recent medication changes, frailty, or previous medication-related adverse events for a comprehensive review.

#### 6.2.2 Interdisciplinary collaboration and responsibility allocation

The application of AI-based medication management requires competent collaboration of pharmacists, physicians, and nurses with IT professionals. The adoption of AI systems may be affected by the differing levels of understanding, trust and acceptance of AI systems by different healthcare professionals (85).

In addition, the issue of responsibility allocation is unresolved. The question of professional liability can never really be ruled out in case the AI-generated warning is ignored and an adverse event occurs, which is related to a medication. To clarify the role of AI recommendations, the governing professional responsibilities, and the documentation that is required need relevant frameworks. In this light, AI should be positioned as a decision-support technology collaborating with the clinician, and not as a clinical decision-maker. It is important for a doctor or nurse to always check the medication decision (83).

### 6.3 Definitions and heterogeneity in polypharmacy research

Another challenge is considerable heterogeneity in the definition and measurement of polypharmacy. Although the term polypharmacy is often used to mean the use of the concurrent use of five or more drugs, some studies use a cut-off of 10 or more drugs, whereas some others look for potentially inappropriate drugs as defined by criteria like the Beers Criteria or the STOPP/START (34).

This inconsistency directly affects the development of AI models, as the definition of the prediction target informs the training labels that are used in supervised learning. When different studies define medication risk using different criteria, AI models are effectively trained to predict different outcomes, making meaningful comparison of model performance difficult. Moreover, inconsistent definitions of outcomes are problematic for evidence synthesis and meta-analysis Disparities in population characteristics, definitions of medication exposure, prediction targets, and outcome measurement have crippled the comparability of the current studies. This is a key barrier to developing generalisable evidence on AI effectiveness in polypharmacy management.

### 6.4 Population-specific considerations

In the older population, the use of various medication and their effectiveness differ along with adverse events. Future AI models from OTC need to be population specific to be efficient.

Presently, data for most AI models is mainly sourced from the general older population. Moreover, there are no customised training frameworks for high-complexity subgroups, including diabetic patients, CKD and older cancer patients. Because drug metabolism characteristics vary significantly by disease state, long-term treatment goals and medication safety risk patterns differ across disease states, general predictive models are unlikely to adequately identify these true clinical risk factors in any specific subgroup.

Patients with diabetes are subjected to long-term use of more than one antidiabetic, antihypertensive and cardiovascular drugs. Such patterns of prescribing are complicated and can lead to an increased risk of hypoglycaemia, falls and ADEs. A more recent study recommended individualised predictive models that can integrate the control of glycaemia, frailty, and patient-centred variables. They found that existing general-purpose AI models may be inadequate to take into account medication safety impacts on long-term fluctuations of glycaemia, functional status, and self-management abilities (9).

Due to complex long-term prescriptions and marked dynamic changes in renal function, the assessment of medication safety risks in CKD patients exhibits strong time-dependence and individual heterogeneity. Consequently, future AI models need to further integrate renal function trajectories, exposure to nephrotoxic drugs, long-term laboratory monitoring data, and information on dynamic prescription adjustments to enhance the ability to accurately identify high-risk CKD patients. Concurrently, dynamic risk modelling that incorporates changes in estimated glomerular filtration rate (eGFR), drug dose adjustments and the presence of multiple comorbidities may facilitate more individualised medication risk prediction and precise medication safety management.

Among older cancer patients, the coexistence of anticancer drugs, supportive care medications and long-term prescriptions for chronic conditions results in a more complex medication burden and potential DDIs. Studies indicate that the prevalence of PIMs is significantly higher in cancer patients than in the general older population, and is closely associated with increased chemotherapy toxicity, functional decline and reduced treatment tolerance (32). Consequently, future AI-driven medication safety management should place greater emphasis on oncology-specific medication risk assessment.

Furthermore, recent research has increasingly suggested that frailty may be a key moderator influencing the progression of polypharmacy risk. Frail older patients are more likely to develop persistent, highly complex medication trajectories, accompanied by a higher risk of functional decline, long-term care dependency and adverse health outcomes (5). Consequently, future AI models need to further integrate patient-centred indicators such as frailty assessment, functional preservation and long-term care dependency, in order to advance precision geriatric pharmacotherapy management toward individualised risk prediction and continuous care.

## 7 Future perspectives

Although AI has demonstrated encouraging predictive capability for medication risk prediction in older adults with polypharmacy, its translation into routine clinical practice remains at an early stage. Future research should shift from retrospective model development toward prospective implementation studies that evaluate whether AI-assisted medication management improves clinically meaningful outcomes, including adverse drug events, medication-related hospitalisations, functional status, health-related quality of life, and mortality.

A key priority will be the development of knowledge-guided AI, in which data-driven algorithms are integrated with established pharmacotherapeutic knowledge rather than relying solely on statistical associations. In future models, assessed clinical assessment tools like the MAI, Beers Criteria, STOPP/START criteria, and anticholinergic burden scales may appear as prior clinical knowledge or weighted model features. Implementing these approaches can enhance the interpretability and acceptability of AI-based recommendations, thereby minimizing the occurrence of technically viable recommendations that lack clinical usefulness.

Another essential direction for research is the integration of multimodal longitudinal data to support dynamic medication risk assessment throughout the patient’s care pathway. In addition to electronic health records and prescription databases, future AI systems may incorporate laboratory trajectories, functional assessments, nursing evaluations, wearable sensor data (e.g., gait, physical activity, and sleep monitoring), and patient-reported outcomes to provide continuously updated medication risk estimates. There is potential for such approaches to enable earlier detection of functional decline, optimisation of medication monitoring intensity, and individualising of deprescribing. Yet, such evidence is limited and currently only from retrospective modelling studies.

AI systems in use today, such as federated learning and large language models, also require further research. Model generalisability may improve in federated learning due to the development of a common model not specific to a single medical institution. In a similar vein, LLMs can help extract information from unstructured medical records that is clinically relevant in nature along with aiding in medication reconciliation and generating medication counselling that focusses on the patient. Although still limited to proof-of-concept studies, retrospective evaluations and early implementation reports, prospective clinical validation of both technologies is lacking.

Future researchers should also look toward implementation science and collaboration among disciplines. The effective use of AI to help with medication management will depend not just on the performance of algorithms, but also on the integration into workflow, acceptance by clinicians and interoperability in the health system. This will require standardised evaluation of the model. Harmonisation of heterogeneous coding systems (e.g., ATC and ICD), external validation across diverse populations, transparent reporting, and appropriate ethical governance will all be essential for successful clinical translation. Ultimately, high-quality multicentre pragmatic clinical trials will be required to determine whether AI-assisted medication management can achieve sustainable improvements in medication safety and patient-centred outcomes for older adults with polypharmacy.

## 8 Conclusion

The current review tackles the predictive role of artificial intelligence in identifying the risk of polypharmacy in older patients as opposed to previous reviews which only described the expanding applications of AI in medication management. The difference is clinically relevant as optimization of deprescribing requires not only the identification of inappropriate medications but also the identification of persons at greatest risk of medication harm before harm occurs. Our review indicates that the key way in which AI in geriatric pharmacotherapy will not replace existing medication assessment models but will rather complement them through dynamic risk stratification based on multi-dimensional clinical data. The process of medication review and preventive intervention can be supplemented using artificial intelligence through the integration of exposure patterns, multimorbidity profiles, lab findings, functional status, and longitudinal care data.

Polypharmacy has become a major challenge in the care of older adults with multimorbidity, contributing to adverse drug events, functional decline, hospitalisation, and increased healthcare utilisation. This review summarises current evidence regarding the application of AI in medication risk prediction and highlights its potential role in identifying potentially inappropriate medications, adverse drug events, medication-related risk trajectories, and high-risk patient populations. AI-based approaches may complement regular medication reviews and support more individualised medication management by integrating information from electronic health records, prescription histories, laboratory data and functional information.

Recent updates indicate that various prediction tasks related to medication use are being accomplished using machine learning, deep learning, graph neural networks and natural language processing. The majority of published studies in this area are retrospective, leading to our caution in interpreting the findings, which mainly evaluate statistical discrimination rather than patient outcomes that are clinically meaningful. Thus, there is sparse evidence that shows the effect on mortality, medication-related hospitalisations, functional status, health-related quality of life.

AI should therefore be considered a clinical decision support tool and not a replacement for pharmacist, physician or nurse medication management. To implement the use of AI-assisted medication management into the regular care of older adults with polypharmacy, we will need several things, including continued collaboration of healthcare professionals with AI, proper external validation, transparency in model development, and high-quality prospective implementation studies.

The clinical utility of AI in polypharmacy management should be evaluated not only on predictive accuracy but also whether it enhances medication safety practices led by pharmacists. In future work, models of human-AI collaboration should be developed where AI gives scalable risk stratification, while the pharmacists give contextual clinical interpretation and patient-centred interventions.
